# Effects and Processes of an mHealth Intervention for the Management of Chronic Diseases: Prospective Observational Study

**DOI:** 10.2196/34786

**Published:** 2022-08-25

**Authors:** Amanda Breckner, Nicola Litke, Linda Göbl, Lars Wiezorreck, Antje Miksch, Joachim Szecsenyi, Michel Wensing, Aline Weis

**Affiliations:** 1 Department of General Practice and Health Services Research Heidelberg University Hospital Heidelberg Germany

**Keywords:** telemedicine, multimorbidity, primary health care, symptom assessment, chronic disease, mobile phone

## Abstract

**Background:**

Mobile health (mHealth) interventions for self-management are a promising way to meet the needs of patients with chronic diseases in primary care practices. Therefore, an mHealth intervention, TelePraCMan, was developed and evaluated for patients with type 2 diabetes mellitus, chronic obstructive pulmonary disease, high blood pressure, or heart failure in a German primary care setting. TelePraCMan entails a symptom diary, an appointment manager, a manager to document goals, and a warning system. The app should foster the self-management of participating patients.

**Objective:**

We aimed to examine the effects of TelePraCMan on patient activation and quality of life and explored the underlying contextual factors, impacts, and degree of implementation.

**Methods:**

In a prospective observational study design, we collected data by using interviews and written questionnaires from participating patients (intervention and control groups) and primary care workers (physicians and practice assistants). The primary outcomes of interest were patient-reported quality of life (12-Item Short Form Survey) and patient activation (patient activation measure). The quantitative analysis focused on differences between patients in the intervention and control groups, as well as before (T0) and after (T1) the intervention. Interviews were analyzed by using qualitative content analysis via MAXQDA (VERBI GmbH).

**Results:**

At baseline, 25 patients and 24 primary care workers completed the questionnaire, and 18 patients and 21 primary care workers completed the follow-up survey. The patients were predominantly male and, on average, aged 64 (SD 11) years (T0). The primary care workers were mostly female (62%) and, on average, aged 47 (SD 10) years (T0). No differences were observed in the outcomes before and after the intervention or between the intervention and control groups. In the additional interviews, 4 patients and 11 primary care workers were included. The interviewees perceived that the intervention was useful for some patients. However, contextual factors and problems with implementation activities negatively affected the use of the app with patients. The main reasons for the low participation were the COVID-19 pandemic and the target group, which seemed to have less interest in mHealth; the interviewees attributed this to the older age of patients. However, the respondents felt that the app would be better accepted in 5 or 10 years.

**Conclusions:**

Although the TelePraCMan app was rated as very good and important by the participants, few patients used it. The digital intervention was hardly implemented and had limited impact in the current setting of German primary care.

**Trial Registration:**

German Clinical Trials Register DRKS00017320; https://tinyurl.com/4uwrzu85

## Introduction

### Background

An increasing number of people have ≥2 chronic conditions (multimorbidity) [1]. Meeting the needs of patients with multimorbidity poses a challenge for health care systems, especially for primary care [2]. Policy makers and health care workers have shown interest in telehealth’s potential for the diagnosis, treatment, and prevention of health problems [3] as well as self-management support [4]. Self-management and self-management support for patients with multimorbidity is complex because of the effects of various diseases and emotional distress. Therefore, innovative care delivery models are required. Mobile health (mHealth) interventions are expected to provide self-management support interventions, which can be tailored to individual needs [5].

Previous research in various patient populations has shown mixed effects of mHealth tools for self-management support [4,5]. A review concluded that through enhanced symptom control, the use of mHealth apps has the potential to improve health outcomes in patients with multimorbidity [5]. A metareview concluded that telehealth is seen as a safe option for the delivery of self-management support, especially for the management of heart failure and type 2 diabetes, but the evidence was inconsistent for other conditions. However, they showed that findings of successful components in the interventions were limited and inconclusive [4]. Another review concerning the combination of mHealth and health coaching for improving self-management in chronic care showed that mHealth and health coaching interventions benefit from each other as well as patients still tend to prioritize human contact. The authors thereby concluded that it is desirable to personalize health technology [6]. A systematic review of reviews also showed that most effective technology-based interventions in improving diabetes self-management combined a feedback loop that connected patients and their health care team using 2-way communication, analyzed patient-generated health data, tailored education, and individualized feedback [7]. The results show that although telehealth interventions enhance self-management, communication and interactions with health care professionals are crucial for patients with chronic diseases, and a combination of both is important.

Furthermore, few studies explored the acceptance and actual use of mHealth interventions by older adults in Germany and the effects of their use on patient-reported outcomes such as patient activation and health-related quality of life. Previous studies on older multimorbid patients have shown that further research is needed for a successful integration of the interventions in patients’ everyday life and in the workflow of primary care practices [8-10].

### Objectives

Therefore, we developed and evaluated the TelePraCMan intervention, which aimed to support the self-management of patients with multimorbidity and enhance their quality of life. We examined the effects of TelePraCMan on patient activation and quality of life and explored the perceptions of practitioners on TelePraCMan. Concomitantly, a process evaluation was undertaken to understand and explore the context, impacts, and implementation process.

## Methods

### Study Design

The developed mHealth app TelePraCMan was tested for 12 months (October 2019 to September 2020) as part of a multicenter randomized controlled study in 10 primary care practices in Baden-Württemberg. Patients, physicians, and practice assistants received a questionnaire at baseline and follow-up. As we did not reach the recruitment targets, we report on the outcome evaluation descriptively. In addition, we conducted a process evaluation in the form of a qualitative interview study and further questions in the questionnaire with these three groups alongside the randomized controlled trial, which is the primary focus of this study. For these reasons, we report the results of the randomized controlled study as well as the process evaluation and refer to the study design as a prospective observational study.

### Recruitment and Sampling

Only practices using the case management program PraCMan [11] for at least 6 months were invited to participate in the study. Approximately 130 practices were contacted via letter, fax, email, or telephone. These practices were selected based on their geographic location and their practice size as well as on the consisting contacts of the research team. First, they were sent a reply form. After the expression of interest, the practices received an information leaflet, a consultation on the phone, and further information documents on the study implementation. After signing a consent form to participate in the study, an appointment was made for a brief training session lasting approximately 90 minutes, during which the study procedure, patient recruitment, and use of the app in the context of care were explained in detail.

The target group of the study was patients enrolled in the case management program PraCMan. These patients had type 2 diabetes mellitus, chronic obstructive pulmonary disease, high blood pressure, or heart failure. Other inclusion criteria were understanding German, being able to give consent, aged >18 years, and having a smartphone or tablet in the household. Eligible patients in the participating practices were addressed by the practice assistants, informed about the study, and asked for their interest.

If the patients were interested in participation, they were informed about the study verbally and in written form and were asked to sign an informed consent form. Patients were randomly assigned to either the intervention or control group. The patients were then given recruitment envelopes that had been prepared for the practices by the study center in Heidelberg. The envelopes included either the documents for use of the app in the intervention group or documents for study participation in the control group. The randomization was performed by lottery within the study center. The envelopes were constructed to look similar in both groups. The practice staff members were not informed about the order. Both envelopes included information leaflets, bank forms for the expense allowance, an initial questionnaire (T0) for the baseline survey, as well as a short information on the next steps for patients and the practice team to simplify study conduction.

The process of patient recruitment was documented by the practice staff in screening lists. The day, the number of patients, and the respective outcome of the recruitment (whether the patient participates) were recorded in these lists. If patients did not wish to participate, the reason for nonparticipation was also recorded.

Of the 130 invited practices, a total of 10 practices with 24 physicians and practice assistants in T0 and 21 in T1 took part in the quantitative study. Of the 141 patients who were asked to participate, 25 completed the quantitative survey in T0 and 18 completed the follow-up survey in T1.

### Intervention

#### TelePraCMan Development

TelePraCMan was developed and programmed by the Department of General Practice and Health Services Research at the Heidelberg University Hospital. In addition to PraCMan, an established model for structured management of chronic diseases in primary care [11,12], TelePraCMan was developed to foster self-management and can be used by practices that regularly use PraCMan and their patients who are subscribed within the PraCMan program. Primary care physicians, medical practice assistants with further training (VERAH [Versorgungsassistent/in in der Hausarztpraxis]), and patients were involved in the development of TelePraCMan via focus groups, interviews, and questionnaires. To adapt the app to the demands of the target group, one survey covered smartphone use and technology affinity to gain knowledge of user requirements before and during the app development process [13]. After evaluating the results, the app was adapted and now contains an appointment manager, a manager for target agreements, and the possibility for general practices to access the symptom data via remote access to TelePraCMan, in addition to the initial basic symptom diaries. TelePraCMan is a web-based app. This means that the app is not installed locally on a user’s smartphone or tablet. Instead, data processing takes place on independent web servers (of University Hospital Heidelberg) and access is realized via a web browser on the user’s smartphone or tablet, which means that an internet connection is required to use the app.

#### TelePraCMan Features

The main features of this app includes symptom diaries for 4 chronic diseases (type 2 diabetes mellitus, chronic obstructive pulmonary disease, high blood pressure, or heart failure). Patients can record values of blood pressure, blood sugar, weight, or mental health conditions in the symptom diaries. Further features of the app included a decent warning system (including what to do and who to contact in an emergency) whenever one of these symptoms or values exceeded the specified thresholds, an appointment manager, a manager for target agreements, and the possibility for primary care practices to access the symptom data via remote access. Within the home page of the app, a daily overview was provided including shortcuts to quickly get to upcoming tasks. The completed tasks were then automatically ticked off within the daily overview. The app was programmed as a browser-based app, so that it did not have to be downloaded and fulfilled the data protection regulations. Some figures of the main features of the app are included in Multimedia Appendix 1.

#### Study Intervention

Patients who were assigned to the intervention group received access to data for using the app in addition to the general study documents. Using a checklist in advance, practice staff and patients determined the frequency and time points at which the patients should document their symptoms or values. Furthermore, the primary care physicians specified thresholds for individualized values (such as the highest or lowest tolerable blood pressure) and documented these in the named checklist. For the first use of the app, the checklist provided guidance on how to individualize the app for themselves.

After the initial setup of the app, patients could use it for 6 months. Patients also continued to receive their usual treatment according to the PraCMan standard guidelines [11]. Before the monitoring appointments, patients could voluntarily transfer the recorded symptoms and values to the practice so that they could be included in the appointments for further treatment planning.

**Figure 1:**
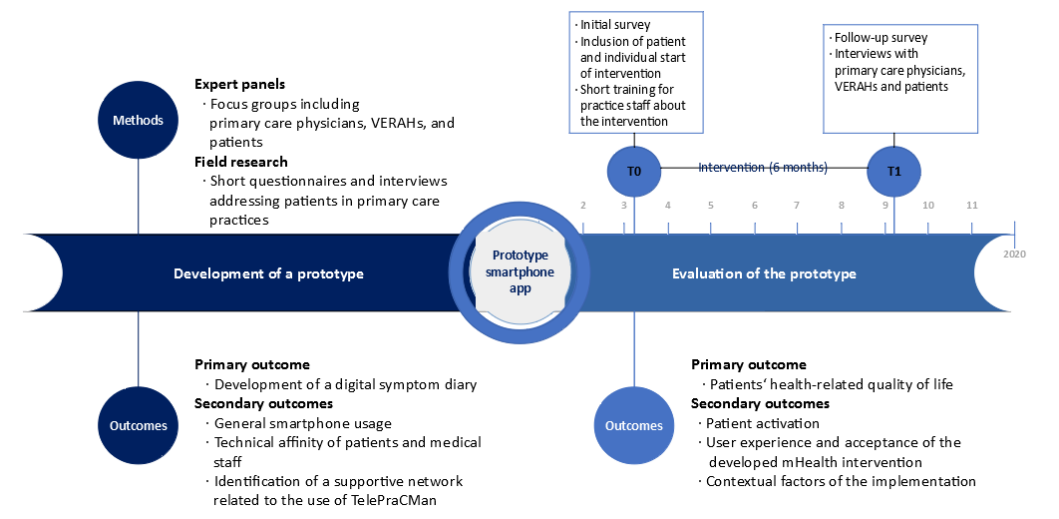
Study overview. VERAH: Versorgungsassistent/in in der Hausarztpraxis. T0: before the intervention; T1: after the intervention.

Patients assigned to the control group continued to receive treatment according to the PraCMan standard care guidelines. In addition to regular monitoring appointments, this also included the use of paper-based symptom diaries to document values and symptoms, which is one key element in PraCMan [11]. Patients in the control group were also included in the study for 6 months. Figure 1 presents an overview of the study.

### Data Collection and Measures

#### Overview

**Figure 2:**
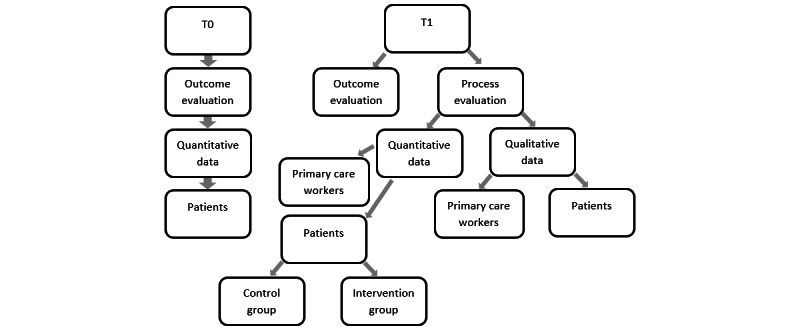
Data collection structure. T0: before the intervention; T1: after the intervention.

Quantitative data were collected before and after the intervention was applied. Data collection at T0 (baseline) took place at the start of the individual intervention for the practices after they had agreed to participate in the study. After the training session, practices received a written survey. Patients also received a written survey at the start of the intervention together with the study documents. Data collection at T1 (follow-up) was performed at the end of the intervention; that is, after 6 months. For practice staff, the T1 survey was administered at the end of the study in September 2020 or after the end of the intervention when the last patient was included. Patients again received a written survey, which was distributed via the practice at the end of the intervention. Figure 2 visualizes the data collection structure.

#### Outcome Evaluation

For the outcome evaluation, we collected data via a written survey in the patient sample at baseline T0 and T1 (follow-up). We measured health-related quality of life using the 12-Item Short Form Survey (SF-12 [14,15]) and patient activation using the patient activation measure (PAM) [16]*.*

The SF-12 consists of 8 subscales that were transformed to a scale from 0 to 100, and the mean value was calculated in each case. In a sample [17], which is representative for Germany, the mean value of each scale is 50 points with an SD of 10 points. To improve comparability of data, the scales were standardized by a z-transformation and then transformed linearly (mean 50, SD 10). Using exploratory factor analysis, the 8 subscales were condensed into two superordinate scales: physical health (physical component summary [PCS]) and mental health (mental component summary [MCS]). The two scales were linearly transformed to a mean of 50 and an SD of 10 [15].

Patient activation was measured using the PAM-13D. Each statement is rated by respondents on a response scale of 1 to 4 (German version), where 1 stands for “disagree strongly” and 4 for “agree strongly.” To calculate the PAM scores (from 0 to 100), the German response options were converted to the standardized metric (0-100). Higher scores indicated that the patient is more activated. On the basis of these scores, the patients were divided into levels. At level 1, patients may not understand their role in making decisions about their health and are more passive. Level 4 patients are able to manage their health on their own but may have problems doing so in stressful situations [16].

#### Process Evaluation

For the process evaluation, we also collected data via the written survey and additional interviews. We measured the evaluation of the TelePraCMan app via the User Experience Questionnaire (UEQ) [18] at T1 in the patient sample. We measured the perceived opportunities and barriers to using TelePraCMan in primary care practices at T0, as well as perception, use, and workload of TelePraCMan in primary care practices at T1 in the primary care worker sample.

Analyzing the user experience data, we used the UEQ Data Analysis Tool version 7 (UEQ Team [19]). The tool calculates the scale means and the mean and SD for each item. It groups the 26 items to create scores for six domains of attractiveness, perspicuity, efficiency, dependability, stimulation, and novelty. The mean scores were calculated for each domain. Values between −0.8 and 0.8 represent a more or less neutral evaluation of the corresponding scale, values >0.8 represent a positive evaluation, and values <−0.8 represent a negative evaluation [18].

A self-administered questionnaire was used to measure the perception and use of TelePraCMan among the practice staff. The questionnaire was based on the technology acceptance model and was tested within the interprofessional study team. It consisted of 21 questions for measurement at T0, which were divided into 5 subscales. The five subscales were as follows: “Perception and use of the VERAH-portal,” “Assessment in relation to the patients concerned,” “Changes brought by TelePraCMan for the patients,” “Workload in the practice,” and “General assessment.” The measurement at T1 consisted of 30 questions, which were divided into 7 subscales. The 5 subscales from the measurement T0 were taken over and extended by the subscales “Ease of use in patients” and “Training”. Each statement was rated by the practice staff from “strongly disagree” (score of 1) to “strongly agree” (score of 5). Mean values were calculated for each subscale, which ranged from 1 (minimum) to 5 (maximum). Values <2.5 stand for a negative evaluation, values of 2.5 to 3.5 for a more or less neutral evaluation, and values >3.5 stand for a positive evaluation.

Additional interviews were conducted with patients and practices after the end of the intervention. The interview guidelines were developed within the project team. Semistructured interviews included questions about the use of the app TelePraCMan, the feasibility of the app, and questions about the integration of the app into everyday (practice) life and about its practicability. All interviews were conducted via telephone between May 2020 and October 2020. The average length of the interviews was 30 minutes (range 13-53). All interviews were digitally recorded with consent of the participants and transcribed verbatim.

### Data Analysis

Descriptive statistics were calculated for all variables included in the quantitative analysis to examine means, SD, distribution for continuous variables, and frequencies for categorical data. For this analysis, we excluded the technological affinity questionnaire and a patient support questionnaire.

To examine changes in mean PAM scores and SF-12 scores before and after the intervention, we conducted a paired *t* test (2-tailed). To examine if there is a difference in the control and intervention groups after the intervention, we conducted an unpaired *t* test. *P*<.05 was considered significant in all analyses. All analyses were performed using SPSS (version 25.0; IBM Corporation).

A deductive-inductive content analysis approach was used to analyze the interview data. A preliminary category system was developed deductively based on the CFIR (Consolidated Framework for Implementation Research [20]) and the interview guide used. To inductively identify additional themes, all transcripts were read thoroughly by 3 members of the research team (AB, NL, and LG). Subsequently, all interviews were coded line-by-line using the deductively formed category system, and additional themes were inductively added where appropriate. The analyses of the three coders were compared and modified if necessary. All data were managed and analyzed using MAXQDA (version 20; VERBI GmbH).

The STROBE (Strengthening the Reporting of Observational Studies in Epidemiology) guideline was used for reporting this study [21].

### Ethics Approval

Ethics approval was obtained from the Medical Ethics Committee of the Medical Faculty of Heidelberg University (S-092/2019) before the start of the study. All participants provided written informed consent before participating in the study. Research conducted in this study was performed in accordance with the Declaration of Helsinki. The study was registered in the German Clinical Trial Register (DRKS00017320).

## Results

### Demography

A total of 27 patients were included in the study, of whom 25 completed a questionnaire at baseline T0 and 18 completed the questionnaire at follow-up T1. Of these 18 participating patients who completed the follow-up survey at T1, 9 (50%) were included in the intervention group, which could use the TelePraCMan app, and 9 (50%) patients were included in the control group (Table 1).

The patients were mostly male (T0: 16/25, 64%; T1: 13/18, 72%), with an average age of 64 (SD 11) years at T0 and 66 (SD 12) years at T1. Most participants stated that they had ≥2 chronic conditions. Most of them were retired (T0: 15/25, 60%; T1: 12/18, 67%) and had a low educational background (T0: 16/25, 64%; T1: 10/18, 56%). Nearly all of them owned a smartphone (T0: 22/25, 88%; T1: 16/18, 89%), which they used in daily life mostly often (T0: 6/25, 24%; T1: 4/18, 22%), or very often (T0: 7/25, 28%; T1: 6/18, 33%; Table 1).

A total of 24 physicians and practice assistants at T0 and 21 at T1 participated in the quantitative study. They were mostly female (T0: 15/24, 62%; T1: 13/21, 62%), with an average age of 47 (SD 10.1) years (T0) and 49.7 (SD 11.45) years (T1; Table 2).

The 4 participating physicians and 7 practice assistants in the additional interviews were, on average, aged 50 years (range 28-73 years) and had been working in the surveyed practices for an average of 20 years (range 6-39 years). The 4 patients interviewed were all retired and, on average, aged 71 years (range 66-78 years).

**Table 1:** Demographics of patients participating in the quantitative survey.

Demographics			Patients at T0 (n=25)		Patients at T1 (n=18)
Age (years), mean (SD; range)			64.25 (SD 11.3; 45-83)		66.00 (SD 11.64; 45-83)
**Sex, n (%)**					
	Female	8 (32)		5 (28)	
	Male	16 (64)		13 (72)	
	No answer	1 (4)		N/A^a^	
**Chronic conditions, n (%)**					
	One chronic condition	7 (28)		5 (28)	
	Various chronic conditions	17 (68)		13 (72)	
	No answer	1 (4)		N/A	
**Marital status, n (%)**					
	Married or cohabiting	14 (56)		11 (61)	
	Unmarried or single	4 (16)		2 (11)	
	Divorced	2 (8)		1 (6)	
	Widowed	4 (16)		4 (22)	
	No answer	1 (4)		N/A	
**Residential situation, n (%)**					
	Living alone	9 (36)		7 (39)	
	Living with others	13 (52)		11 (50)	
	No answer	3 (12)		2 (11)	
**Number of inhabitants in the residence, n (%)**					
	<5000 inhabitants	1 (4)		N/A	
	Between 5000 and 20,000 inhabitants	11 (44)		8 (44)	
	Between 20,000 and 100,000 inhabitants	2 (8)		N/A	
	Over 100,000 inhabitants	9 (36)		9 (50)	
	No answer	2 (8)		1 (6)	
**Educational level, n (%)**					
	High educational level	2 (8)		2 (11)	
	Middle educational level	2 (8)		2 (11)	
	Low educational level (eg, elementary school)	16 (64)		10 (56)	
	No school-leaving qualification	3 (12)		3 (17)	
	No answer	2 (8)		1 (6)	
**Employment situation, n (%)**					
	Working	5 (20)		3 (17)	
	Retired	15 (60)		12 (67)	
	Not economically active	4 (16)		3 (17)	
	No answer	1 (4)		N/A	
**Owner of a smartphone, n (%)**					
	Yes	22 (88)		16 (89)	
	No	3 (12)		2 (11)	
**Using the smartphone in daily life, n (%)**					
	Seldom	4 (16)		3 (17)	
	Sometimes	5 (20)		3 (17)	
	Often	6 (24)		4 (22)	
	Very often	7 (28)		6 (33)	
	Missing	3 (12)		2 (11)	
**Group allocation, n (%)**					
	Intervention group	N/A		9 (50)	
	Control group	N/A		9 (50)	

^a^N/A: not applicable.

**Table 2:** Demographics of physicians and practice assistants participating in the quantitative survey.

Demographics		Physicians or practice assistants at T0 (n=24)	Physicians or practice assistants at T1 (n=21)
**Professional qualification, n (%)**			
	Physician	11 (46)	8 (38)
	Practice assistant	13 (54)	12 (57)
	No answer	0 (0)	1 (5)
Age (years), mean (SD; range)		47.14 (10.1; 27-65)	49.74 (11.45; 29-74)
**Sex, n (%)**			
	Female	15 (62)	13 (62)
	Male	9 (37)	7 (33)
	No answer	0	1 (5)
**Type of medical practice, n (%)^a^**			
	Single-handed practice	4 (36)	N/A^b^
	Joint practice	6 (55)	N/A
	Other	1 (9)	N/A
**Number of inhabitants in the location of the medical practice, n (%)^a^**			
	<5000 inhabitants	1 (9)	N/A
	Between 5000 and 20,000 inhabitants	8 (73)	N/A
	Between 20,000 and 100,000 inhabitants	N/A	N/A
	>100,000 inhabitants	2 (18)	N/A

^a^Numbers are referring to physicians.

^b^N/A: not applicable.

### Outcome Evaluation

In 10 primary care practices, a total of 141 patients with multimorbidity were asked to participate in the study. Of these 141 patients, 114 (80.8%) did not want to participate in the study. The reasons for nonparticipation were mainly that patients did not own a smartphone (53/114, 46.5%), had no interest in participation (23/114, 20.2%), or had no access to the internet (16/114, 14% Table 3).

At date T0, the mean MCS-12 score was 44.1, with a median of 43.1 across all respondents, ranging from 29.03 to 61.17. At T1, the mean MCS-12 score (39.3) was slightly lower, with a median of 39.7 across all patients, ranging from 29.81 to 57.58. On average, all participants had a PCS-12 score of 36.8, with a median of 36.7, ranging from 25.73 to 54.60. Slightly higher, at date T1, the mean PCS-12 score was 39.9, with a median of 37.3, ranging from 30.73 to 54.45 (Multimedia Appendix 2).

**Table 3:** Reasons for not participating in the study named by patients and documented by practice assistants (N=114).

Reasons for nonparticipation in the study^a^	Value, n (%)
Technical requirement not met: no smartphone available	53 (46.5)
No interest	23 (20.2)
Technical requirement not met: no internet accessible	16 (14)
Mentally unable	13 (11.4)
No time	5 (4.4)
Language problems	4 (3.5)
Physically unable	4 (3.5)
Need personal contact	1 (0.9)
Spouse is responsible for technical and devices	1 (0.9)

^a^Multiple answers were possible.

Across all respondents, the mean PAM score was 77.9, with a median of 76.9 at date T0, ranging from 28.2 to 100. More than half (14/21, 56 %) of all participants had patient activation scores of ≥72.5. Only 1 participant reported lower activation scores at level 1. The PAM score across all patients at date T1 was 82.0, with a median of 82.0, ranging from 61.54 to 100. More than a quarter (14/16, 78%) of the participants had patient activation scores of ≥72.5. None of the patients reported lower activation scores for levels 1 or 2 (Multimedia Appendix 2).

There was no significant difference in outcome measures SF-12 scores and PAM scores before and after the intervention as well as in the comparison of the intervention and control groups (Multimedia Appendix 2).

### Process Evaluation

The overall perception of physicians and practice assistants regarding TelePraCMan was neutral at the beginning (T0). The mean score of the perception of TelePraCMan was 3.6, with a median of 3.6 (SD 0.33) across all practices, ranging from 3.05 to 4.52. Looking at the topics of perception in detail, the use and help of the VERAH-Portal were evaluated as the best (mean 4.5, SD 0.34), whereas the perception of the amount of work was evaluated as the worst (mean 2.7, SD 0.73; T0). After using TelePraCMan for half a year (T1), the overall perception was still neutral and slightly worsened (mean 3.3, SD 0.56). The best evaluated topic was again the use and help of the VERAH-Portal (mean 4.3, SD 0.78), whereas the worst evaluated topic was the use in the patients (mean 2.8, SD 06). In the free text entries, practices expressed their perception of older adult patients who need primary care and continuity and still tend to feel a general rejection toward digitalization (Table 4).

**Table 4:** Perception and use of TelePraCMan among physicians and practice assistants.

			Physicians or practice assistants at T0 (n=24)		Physicians or practice assistants at T1 (n=21)
**Perception and use of TelePraCMan (score), mean (SD)**					
	VERAH^a^-Portal and practice computer	4.5 (0.34)		4.3 (0.78)	
	Use in target patients	3.0 (0.54)		2.8 (0.60)	
	Changes for patients	3.7 (0.40)		3.2 (0.80)	
	Amount of work (VERAH or physicians)	2.7 (0.73)		3.3 (1.32)	
	General assessment for VERAH or physicians	3.8 (0.79)		4.5 (0.81)	
	Easy use	N/A^b^		2.9 (1.10)	
	Training	N/A		3.6 (0.89)	
Overall score (all items), mean (SD; range)			3.6 (0.33; 3.05-4.52)		3.3 (0.56; 2.50-4.37)

^a^VERAH: Versorgungsassistent/in in der Hausarztpraxis.

^b^N/A: not applicable.

Evaluating the UEQ, the 8 patients in the intervention group evaluated TelePraCMan as mainly positive. The mean score in the aspect of attractiveness was 1.39 (SD 0.76), in the aspect of efficiency it was 0.87 (SD 1.12), in the aspect of dependability it was 1.12 (SD 0.76), and in the aspect of stimulation it was 1.13 (SD 0.57). Only the aspects of perspicuity and novelty were evaluated as neutral. The mean score in the aspect of perspicuity was 0.71 (SD 0.85) and in the aspect of novelty it was 0.75 (SD 0.73).

### Interviews

In the interviews, we found that TelePraCMan was perceived as useful for some patients. However, contextual issues and problems with implementation negatively affected the use of the app with patients. Overall, the app and the entire project were rated as very good and important, respectively, by the respondents. However, the respondents agreed that the app would be well accepted in 5 to 10 years, as now the patients who need care still tend to feel a general rejection toward digitalization.

### Implementation Activities

Regarding the implementation activities, physicians and practice assistants described the initial training as good and clear. The launching at the practice computers as well as the dealing with the VERAH-Portal were unproblematic. It was described as being easy and user-friendly.

In contrast, recruiting patients for this study was described as difficult and tedious. Practices reported on the COVID-19 pandemic as a reason for low participation of patients. Patients were rarely visiting the practice and it was less time for recruiting owing to other priorities concerning practice management in this time. Nevertheless, the narratives also focused on the fact that the wrong target group was being addressed as a reason for the low participation. Primary care workers reported that PraCMan mainly includes “high-risk” patients with multimorbidity who are commonly old, who often do not own a smartphone, or do not have access to the internet (see the Outcome Evaluation section in Results for reasons for nonparticipation). Practices reported that this group of patients was not interested or had a general rejection of digitalization. The use of smartphones or the internet was not commonplace in this target group:

Then,...came the Corona period. So, our VERAH...assured me that they addressed patients and then had the experience that those they addressed did not react positively to it, i.e., that it was not possible to convey what it was about or that a device was not even used or available. So, to say, the affinity for technology of those addressed was close to zero. But there were only four or five patients that we addressed and then it was Corona chaos anyway.... It was so that the dominance of the urgent pushed it into the background.

Physician 1

The implementation and setup on the patients’ smartphones were mostly done together with practice assistants or friends and family. Technical problems within the first registration of the patients were observed, which could mostly be solved after consultation of the practice assistants or a project team member.

### Patients’ Individual Context: Facilitators and Barriers

Barriers to the use of TelePraCMan were, in the view of the respondents, in some cases too much effort for the patients to learn how to use the app or because of the financial situation of some patients that they could not afford a smartphone. Moreover, they mentioned technical problems such as a mobile phone that was too old, problems with registration or a virus on the smartphone. A lack of patient compliance, a lack of acceptance, or a lack of skills was also in the forefront of interviewees’ minds:

It was very complicated for those who could actually do it. You could tell that they were maybe a bit familiar with the mobile phone and that they might be able to send a message, but it was difficult to use a special app. So, I think young people up to 60 are fit, not necessarily up to 60. The young people who have just grown up with these smartphones, who know how to use apps.... There are things like health records or video conferences that you can do with your mobile phone. But for those who need a video conference because they have difficulty walking or are multimorbid, they can’t use it. So, I see that as difficult, and everyone wants that. They used to get by without a mobile phone, why should they want to use a mobile phone now? If I explain to them that we can communicate with them, then there is actually an app, or there is a mobile phone and there is an app, and we can communicate with them, no, the older people actually want to have personal contact.

Practice assistant 1

Patients’ previous experience of using similar apps and a support network in case of technical problems were mentioned as beneficial for them and their use of TelePraCMan. Patients mentioned the simple condition of the app, which did not take much time for them. Patients also articulated enjoying the use of the app:

It may not be the same for everyone, but I had no problem with it at all and honestly, I was pleasantly surprised by the program. It was good, I enjoyed it.

Patient 1

### Impact

Different factors were mentioned regarding the impact of TelePraCMan on health care. The only negative aspect mentioned was that the physician-patient relationship could possibly deteriorate because of the personal contact and the “emotionality” that might be lost. However, one patient also found it positive that small things occurred in the app that can be discussed over the phone; for example, concerning medication.

Physicians and practice assistants also said that TelePraCMan can increase and improve the physician-patient relationship because of the fact of talking on the same information basis. The shared information could also partially change the communication by better involving the patient in the monitoring. However, some practice assistants and physicians did not perceive any changes in communication or in the monitoring appointments.

The release of data to the practice was seen as positive by all respondents. In this context, an aspect that was frequently valued was the quick detection and reaction to situations that require treatment or an intervention from a long distance. Furthermore, respondents described that the app could gain relevance in pandemic situations such as the COVID-19 pandemic because it helps to care for chronically ill patients outside the practice:

And then the person who can react to this has all the values from me and just these two three keywords that I have given to the computer in the evening via TelePraCMan, which can then be looked at by anyone who is important, so that it is not forgotten afterwards.

Patient 2

Interviewees also supported that TelePraCMan motivated patients and thus promoted self-management and informed them about the actual state of their own health:

Right, for me it was always interesting because I could always see where I stood every week. I knew exactly when I had sinned, what I had done with my cholesterol or my sugar levels, and that was interesting.

Patient 1

Another positive effect mentioned was that it relieved the workload of the practice assistants and physicians, as the values were directly accessible to them and did not have to be requested by the patient:

Often it’s like this, you call the patient, and then the patient says “Oh yes, I’m doing so well,” and then you start asking a little bit and then and “Ah yes right, there was something once.” I think it would possibly also shorten the time, because you have everything at a glance, you see it, ah ok this and that happened, good, and you can deal with it straight away. And often it’s like this, you’re on the phone with the patients and you spend an incredibly long time...because you have to pull everything out of their noses. Not that they don’t notice that they don’t want to tell me, maybe it just doesn’t occur to them at that moment. And so, everything would just be listed, it would all be there, this and this and this and this has already happened, the blood pressure was like this and I think that would make the work a lot easier.

Practice assistant 2

Suggestions for improvement from the respondents were the possibility to enter the values more flexibly in terms of time, a simpler setup and registration, and that the values can be called up in the long term. Enhancements to the app were mentioned in terms of a graphical representation of the values, a pedometer, and a food diary, as well as the inclusion of the medication plan and a plan of what needs to be done if a threshold (eg, blood pressure) is exceeded.

## Discussion

### Principal Findings

First, the participation rate (27/141, 19.2%) was low in this study. Documentation and respondents in the interviews traced it back to the COVID-19 pandemic, as many patients asked to participate did not have an appropriate smartphone or had no interest in participation. Our quantitative findings indicate no effects on patient activation or health-related quality of life. However, the user experience of patients using the app was mainly positive. Practitioners’ perceptions of TelePraCMan was predominantly neutral. Within the qualitative study, we found that the interviewees perceived that the intervention was useful for some patients. However, contextual issues and problems with implementation negatively affected the use of the app with patients. Overall, the app and the entire project were rated as very good and important by the respondents and will be more accepted in recent years.

According to primary care workers, the COVID-19 pandemic had an impact on recruitment and the low participation rate. However, recruitment took place from November 2019 to March 2020; therefore, only the beginning of the pandemic was part of the recruitment time. Thus, the low participation rate may be due to an interaction of different factors. The primary care workers’ narrative as well as the documentation of the reasons for nonparticipation revealed that the addressed target group showed other needs, as older adults commonly do not use smartphones as much as younger people. In terms of figures, 46% (53/114) of the nonparticipating patients did not own an appropriate smartphone. However, the respondents agreed that the app will be well accepted in 5 to 10 years, as now the patients who need the care still tend to feel a general rejection toward digitalization.

### Comparison With Prior Work

mHealth interventions such as TelePraCMan are often not used in the “real” world [22]. Therefore, it is crucial to check the development and implementation for understanding and learning for future digital projects. For this purpose, the person-based approach for digital interventions from Yardley et al [23] can be used. The approach reflects the four stages: planning, design, development and evaluation of acceptability and feasibility, and implementation and trialing of an intervention; and offers a systematic means of addressing the user experiences.

The target of the planning phase is to identify the key behavioral needs and issues the challenges have to address, mainly via qualitative research [23]. Within TelePraCMan, primary care physicians, VERAHs, and patients were involved in the development of TelePraCMan via focus groups, interviews, and questionnaires to gain insights to their wishes and needs [13].

In the design phase, it is crucial to create guiding principles for the developers with the features of the intervention [23]. Within TelePraCMan, the evaluations of the previously named data sources were combined in the project team, and a concept for the app was derived. This concept was first translated into a paper prototypes. These paper prototypes served as the basis for further short interviews with patients and teams in primary care practices. These results were then used to design the app in the form of so-called “action sheets.” These comprised relevant features, characteristics of the target group, potential barriers and facilitators, and possible variations for each page of the app. This process allows apps to be tailored to the specific target group [24]. The action sheets were given as a list of requirements to the computer scientists who programmed a prototype of the app based on them. Throughout the process, all scientists and programmers worked closely together. In addition, primary care physicians were involved in the process as experts.

In the development and evaluation phases of acceptability and feasibility, the intervention components should be evaluated and optimized from the user perspective via user reactions to every intervention element and detailed longitudinal mixed methods case studies. Within TelePraCMan, the app prototype was initially tested by employees of the department. After appropriate adjustments, the prototype was tested during an advanced training course of the department with primary care physicians and VERAHs. These test runs were performed without initial explanations or instructions to enable the most intuitive handling. Comments, questions, uncertainties, or technical problems of the test runs were recorded in writing, evaluated, and adjusted in the app together with the computer scientists. With regard to the person-based approach, we realized the analyzing of the user reactions but did not perform an iterative cycle moving between user feedback and changes to the intervention and did not perform longitudinal case studies where the target group could use the app at a certain time on their own. Performing these two aspects may have led to improvements of the app and insights into how the target group of patients with chronic diseases perceive and use the app. We might also gain insights into their internal motivation for using the app and may gain ideas of the target group to motivate other people to participate in the study.

In the implementation and trialing phase, the intervention should be evaluated in real life via mixed methods process analyses to identify further modifications for future implementations [23]. Within TelePraCMan, a randomized controlled trial and a process evaluation were performed, and the results are included here and discussed in comparison with prior work.

Although data from the German Federal Statistical Office showed that the number of people owning a smartphone declines with increasing age [25], the latest survey from the German Federal Statistical Office [26] concerning the use of information and communication technologies in private households showed that in extrapolations, 10,683 of 16,640 (64.2%) people aged ≥65 years used the internet for private concerns. Of these 10,683 people, 74% (7905/10683) used a smartphone to access the internet for private concerns, but only 3% used devices connected to the internet for monitoring of blood sugar, blood pressure, or weight [26]. Nevertheless, the data from the German Federal Statistical Office showed that older adults are not commonly averse to use smartphones. In contrast, primary care workers in our study raised concerns that digitalization is widely implemented in the health care sector; still, there are people who need the interventions the most but are not able or not ready to use mHealth interventions. It remains open how to deal with and meet the concerns of this patient group in relation to eHealth and mHealth. Addressing their concerns during the recruitment phase may have enhanced participation.

A study by Steele Gray et al [27] evaluating a tool of electronic patient-reported outcomes in a 4-month trial showed no changes in outcomes of patient activation and quality of life, which was traced back to the small sample size of the study. The authors also explored factors for nonparticipation, these were mainly that patients were overwhelmed with the management of their diseases and patients did not want to add another responsibility, unawareness of having health goals they could facilitate, no self-identification with having a chronic condition, and only in fourth place concerns and less experience with technology [27]. In contrast, the most frequent reason for nonparticipation in our study was the unavailability of an appropriate smartphone or internet access as well as uninterest in participation. The unavailability might be an ostensible reason for patients, so they do not have to tell that they are already overwhelmed with their management of their diseases. It is questionable whether the use of smartphones in our study would have increased participation. A previous study with potential users of TelePraCMan found that older patients with multimorbidity preferred personal support over internet-based support [13]. In every case, the information on the benefits of using an mHealth tool could be facilitated for older patients. This was also found in a larger trial concerning a telehealth service with regular phone calls and standardized scripts, where 609 patients with depression and 641 patients with cardiovascular disease were recruited, showing only modest effect for self-management [10]. In an embedded qualitative study with practitioners and patients, they found that contextual issues in patients’ lives, such as motivation to improve their health or the interest in the intervention, as well as some problems with implementation reduced the impact. Furthermore, the authors concluded that to enhance patient engagement in telehealth-motivated staff that offer the intervention with continuity is a crucial factor. Moreover, the intervention should be tailored to individual patients’ needs as well as the content, time required, and benefits should be clearly communicated to potential users [10].

Another crucial point discussed in the interviews was the possible loss of the physician-patient connection and the influence on communication. Our findings are supported by a study with nurse practitioners, which found that they believed that it is difficult to communicate by telehealth owing to difficulty in perceiving nonverbal signals. They concluded that interpersonal communication should be a part of their professional training [28]. Some participants in our study feared the loss of the connection, whereas others pointed out that the app could facilitate communication since patients and providers are talking on the same information basis. The aim of the study was not to replace consultations but to make it easier for patients to spend time between appointments, so only the information basis changes, which was described as positive from respondents. A study from China found that face-to-face patient-provider communication had a positive and direct effect on web-based patient-provider communication at a later point. In addition, patient trust and patient satisfaction had a positive impact on the relationship between face-to-face and web-based patient-provider communication [29]. This could also apply to our participants as chronically ill patients who often have a strong connection with their primary care physicians.

We also collected data on practitioners’ perceptions on the app. We found that after using TelePraCMan for half a year, the overall perception was neutral. Primary care workers evaluated the use and help of the portal where they could see patients’ data the best but were skeptical for the use of patients. Our findings are supported by a systematic review on factors that could facilitate or act as a barrier for health professionals to use mHealth in their work. They found various factors at the individual, organizational, and contextual levels associated with the use of mHealth tools. The most important factors were usefulness and ease of use of the app in patients [30].

### Strengths and Limitations

One strength of this study was the inclusion of different perspectives via primary care physicians, practice assistants, and patients using the intervention in both parts of the study. One of the most important limitations in our study, which we already named, is the small sample size in the quantitative part, which was only partly contingent on the COVID-19 pandemic. Owing to the small sample size, the values had to be interpreted very carefully, as the coefficients may result solely from sampling effects. Furthermore, transferability and comparability with other samples may not be possible. However, the data from the process evaluation served as a possible explanation for the outcome evaluation and provided deeper insight.

### Conclusions

This prospective observational study is one of the first studies concerning an mHealth intervention for chronically ill patients in a primary care setting in Germany. The app TelePraCMan was developed involving physicians, practice assistants, and patients and was adapted to their demands. However, this study showed that it was hardly implemented. Owing to the small sample size, the effects on patient activation and quality of life could not be determined. Currently, this app may be a support system for only a few patients in the target group. Future interventions should facilitate the information on the benefits of using an mHealth tool for older patients, and it is crucial to involve motivated staff who offer the intervention with continuity to the patients. Overall, the app and the entire project were rated as very good and important by the participants. However, the respondents agreed that the app would be well accepted in 5 to 10 years, as now the patients who need the care still tend to feel a general rejection toward digitalization.

## Appendix

Multimedia Appendix 1 Screenshots of TelePraCMan.

Multimedia Appendix 2 Results of the outcome measures and comparison of patient outcomes before (T0) and after (T1) the intervention and between the intervention and control group.

## Abbreviations

CFIR: Consolidated Framework for Implementation Research
MCS: mental component summary
mHealth: mobile health
PAM: patient activation measure
PCS: physical component summary
SF-12: 12-Item Short Form Survey
STROBE: Strengthening the Reporting of Observational Studies in Epidemiology
UEQ: User Experience Questionnaire
VERAH: Versorgungsassistent/in in der Hausarztpraxis
