# Supplementary material for: Effects and Processes of an mHealth Intervention for the Management of Chronic Diseases: Prospective Observational Study
Source: JMIR Form Res. 2022 Aug 25;6(8):e34786. doi: 10.2196/34786 (PMC9459841; doi:10.2196/34786)

Figures of TelePraCMan

Figure 1: Main menu


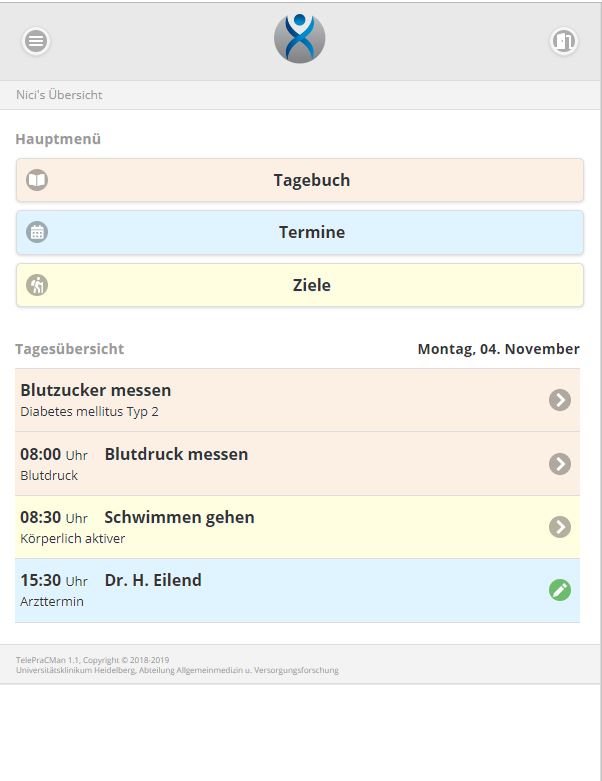


Figure 2: Editing the symptom diary


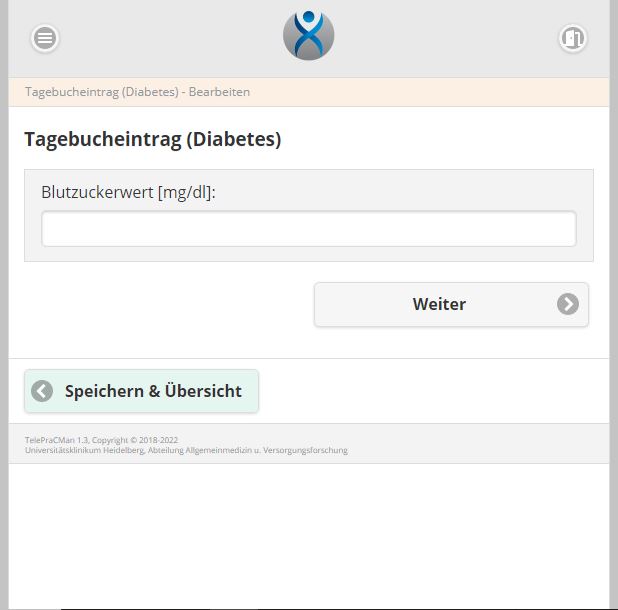


Figure 3: Editing the goals


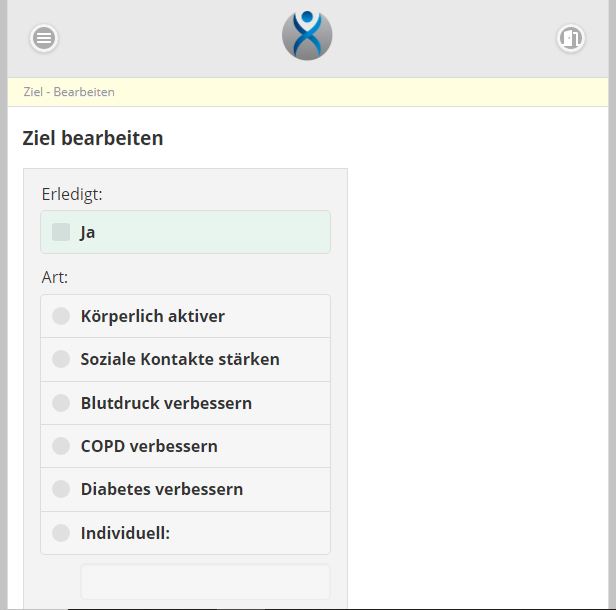


Figure 4: Analysing of symptoms for patients with high blood pressure


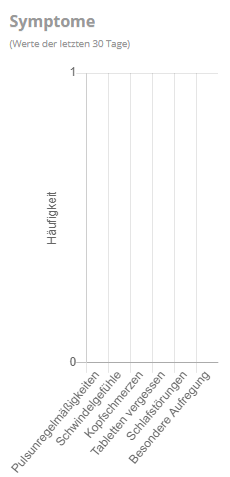

Supplement: Multimedia Appendix 1 [file formative_v6i8e34786_app1.docx]
