# Supplementary material for: Effects and Processes of an mHealth Intervention for the Management of Chronic Diseases: Prospective Observational Study
Source: JMIR Form Res. 2022 Aug 25;6(8):e34786. doi: 10.2196/34786 (PMC9459841; doi:10.2196/34786)
Supplement: Multimedia Appendix 2 [file formative_v6i8e34786_app2.docx]

Supplemental Material - Effects and processes of a mHealth intervention for management of chronic diseases – prospective observational study

Table: Outcome measures and comparison of patient outcome between T0 and T1; between intervention and control group

|  | **T0**  **Patients**  **n=21 (100%)** | **T1**  **Patients**  **n=16 (100%)** | **T (df)** | **P** |
| --- | --- | --- | --- | --- |
| **SF12 Scores** mean (SD) | | | | |
| mental component score (MCS-12) | 44.1 (9.49)  Min 29.03 Max 61.17 | 39.3 (7.61)  Min 29.81 Max 57.58 | 1,78 (13) | 0.097 |
| physical component score (PCS-12) | 36.8 (7.69)  Min 25.73 Max 54.60 | 39.9 (7.17)  Min 30.73 Max 54.45 | -0.83 (13) | 0.418 |
| **PAM Score** mean (SD) | | | | |
|  | 77.9 (17.35)  Min 28.21  Max 100.0 | 82.0 (13.30)  Min 61.54  Max 100.0 | -0.97 (17) | 0.343 |
| **PAM Level** n (%) | | | | |
| Level 1 | 1 (4.0) | 0 |  |  |
| Level 2 | 0 | 0 |  |  |
| Level 3 | 10 (40.0) | 4 (22.2) |  |  |
| Level 4 | 14 (56.0) | 14 (77.8) |  |  |
|  |  |  |  |  |
|  | **Control group**  **n=7** | **Intervention group**  **n=9** | **T (df)** | **P** |
| **SF12 Scores** mean (SD) | | | | |
| mental component score (MCS-12) | 40.1 (7.4) | 38.7 (8.1) | 0.34 (14) | 0.737 |
| physical component score (PCS-12) | 39.5 (5.9) | 40.1 (8.3) | -0.15 (14) | 0.880 |
| **PAM Score** mean (SD) | | | | |
|  | 84.0 (15.26) | 80.0 (11.59) | 0.62 (16) | 0.541 |
